# A survey exploring the practices of smoking cessation support among hospital-based healthcare providers

**DOI:** 10.1186/s12913-023-09657-4

**Published:** 2023-06-16

**Authors:** Ingeborg Farver-Vestergaard, Peter Hjorth, Charlotta Pisinger, Pia Veldt Larsen, Anders Løkke

**Affiliations:** 1 Department of Medicine, Lillebaelt Hospital, Beriderbakken 4, Vejle, 7100 Denmark; 2 Department of Regional Health Research, University of Southern Denmark, Odense, Denmark; 3 Psychiatric Hospital, Region of Southern Denmark, Vejle, Denmark; 4 Center for Clinical Research and Prevention, Capital Region of Denmark, Bispebjerg-Frederiksberg University Hospital, Copenhagen, Denmark; 5 Danish Heart Foundation, Copenhagen, Denmark; 6 Faculty of Health and Medical Sciences, Department of Public Health, University of Copenhagen, Copenhagen, Denmark; 7 Mental Health Services, Region of Southern Denmark, Vejle, Denmark

**Keywords:** Change management, Health and safety, Organisation of health services, Quality in health care, Public health

## Abstract

**Background:**

Hospital visits constitute a ‘window of opportunity’ for initiating smoking cessation attempts, and healthcare providers (HCPs) play an important role in supporting patients to stop smoking. Yet, the current practices of supporting smoking cessation in the hospital setting are largely unexplored. The aim of this study was to explore practices of smoking cessation support among hospital-based HCPs.

**Methods:**

HCPs working in a large hospital in the secondary care sector completed an online, cross-sectional survey, including sociodemographic and work-related factors as well as 21 questions assessing practices of smoking cessation support based on the “five As” framework. Descriptive statistics were computed, and predictors of HCPs giving patients advice to stop smoking were explored using logistic regression analysis.

**Results:**

All employees (N = 3998) in the hospital received a survey link; 1645 (41.1%) HCPs with daily patient contact completed the survey. Smoking cessation support in the hospital setting was limited with regard to assessment of smoking; providing information and advice; planning and referral for further support; and follow-up on smoking cessation attempts. Almost half (44.8%) of participating HCPs with daily patient contact never or rarely advise their patients to stop smoking. Physicians were more likely than nurses to advice patients to stop smoking, and HCPs in outpatient clinics were more likely to give advice than inpatient clinic HCPs.

**Conclusion:**

Smoking cessation support is very limited in the hospital-based healthcare setting. This is problematic, as hospital visits can be windows of opportunity to help patients change their health behaviour. An intensified focus on the implementation of hospital-based smoking cessation support is needed.

**Supplementary Information:**

The online version contains supplementary material available at 10.1186/s12913-023-09657-4.

## Introduction

Tobacco smoking is a major risk factor for the development and progression of somatic, as well as mental, illness [1–3]. A considerable proportion of patients continue smoking after the diagnosis of a mental or a somatic illness, which is associated with suboptimal treatment outcomes and reduced quality of life [3–5]. Therefore, smoking cessation is a highly important and cost-effective element of inpatient and outpatient healthcare [6], and initiatives for smoking cessation support are described in recently updated guidelines from the National Institute for Health and Care Excellence in the UK [7]. Furthermore, being diagnosed with and receiving treatment for a severe illness has been described as a ‘teachable moment’, when patients’ motivation to quit smoking is increased [8]. For example, up to 70% of smokers with lung cancer are interested in quitting [9], and patients newly diagnosed with heart disease or asthma have increased quit rates, compared to healthy individuals [10].

Nonetheless, many motivated patients do not succeed in quitting and/or maintaining their attempt to stop smoking due to their strong biopsychosocial addiction, for example nicotine addiction, using smoking to manage feelings of distress, and being part of a social group of smokers [11]. Therefore, the vast majority of patients, who are motivated to quit smoking would benefit from support from healthcare providers (HCPs) [12]. Recent evidence suggests that persistent smoking cessation is most effectively achieved with a combination of behavioural support and pharmacological treatment (i.e. nicotine substitution, varenicline or bupropion), which increases the chances of prolonged abstinence by almost fourfold (relative risk 3.88, 95% confidence interval [CI] 3.35–4.6), compared to usual care [13, 14]. Smoking cessation support can be delivered in the form of short interventions during a patient’s conventional treatment plan or as a separate add-on programme with several individual or group-based meetings [15–17]. A Cochrane review has found that smoking cessation interventions that begin during a hospital stay and that are continued after discharge increased smoking cessation rates significantly after discharge (risk ratio 1.37, 95% CI 1.27–1.48) [18]. Yet, the optimal timing of smoking cessation advice is highly individual, and repetitive advice from different types of HCPs (e.g., nurses, physicians, healthcare assistants) at different points in the care trajectory is needed [19–21].

Despite its efficiency, the implementation of smoking cessation support in healthcare settings is limited. For example, in an American registry-based study, only 36% of lung cancer patients received smoking cessation advice [9], and results from the Eurobarometer surveys show that, at the population level, the use of evidence-based cessation methods in European countries is low and declining [22]. Beliefs and attitudes among HCPs appear to be an important barrier in this regard. A systematic review of general practitioner (GP) beliefs and attitudes towards discussing smoking cessation with patients found that a significant minority of GPs hold beliefs and attitudes that are unlikely to promote smoking cessation counselling [23]. Forty-two per cent of physicians reported that discussing smoking was too time-consuming, and 38% believed that discussing smoking with patients was ineffective. Moreover, 22% lacked confidence in their ability to discuss smoking cessation, and 18% felt uncomfortable doing so. A mixed-methods systematic review indicated that more than 40% of mental health providers reported barriers to and negative attitudes in discussing smoking cessation with their patients [24]. The more commonly held belief was that patients are not interested in or capable of quitting. Furthermore, qualitative results revealed that smoking was perceived as a cultural norm; an important coping mechanism for patients; and a useful tool to establish a therapeutic relationship [24].

Taken together, HCP practices related to smoking and smoking cessation may undermine the implementation of efficient smoking cessation interventions. The existing knowledge in this area is based on relatively small studies of HCPs from different healthcare settings, and there are no previous studies based on a representative sample of HCPs in the hospital setting. Therefore, the aim of the present study was to explore the practices of smoking cessation support among HCPs in an entire hospital.

## Methods

An online, cross-sectional survey was performed by researchers from the Department of Medicine, Lillebaelt Hospital, Vejle, and the Psychiatric Hospital, Vejle, Denmark. The study adhered to the Checklist for Reporting Of Survey Studies (CROSS) [25]; see Supplementary Material 1).

The survey was designed using the Danish web-based survey system ‘SurveyXact’ (http://www.surveyxact.dk) and distributed among HCPs employed at a large hospital in the Region of Southern Denmark, covering a total of four geographical locations: Vejle, Kolding, Svendborg and Middelfart. Participation in the survey was voluntary and anonymous. Study procedures were approved by the hospital management, and the processing of personal data was approved by the Region of Southern Denmark and listed in the internal record prior to the initiation of data collection.

### Survey items

The survey items were designed by the research team based on existing studies [26, 27] (see Supplementary Material 2). The subset of survey items used in the present analyses are described below.

#### Sociodemographic and work-related variables

Participants were asked to report their age and sex, and a number of work-related variables were assessed, including years of healthcare experience; department type (somatic vs. psychiatric); type of clinic (outpatient clinic; inpatient bed unit; accident and emergency [A&E]; intensive care unit; other); and type of HCP (nurse; physician; healthcare assistant; physiotherapist; occupational therapist; psychologist; social worker; pedagogue; student; other).

#### Practice of smoking cessation support

A total of 21 items in the survey measured practices of smoking cessation support based on the ‘five As’ framework (Ask, Advice, Assess, Assist, Arrange), as applied in a previous study [27]. Items were rated on 5-point Likert scales (1 = ‘never/almost never’; 2 = ‘rarely’; 3 = ‘sometimes’; 4 = ‘often’; 5 = ‘always/almost always’).

### Analysis

All analyses were performed with SPSS Statistics Version 28.0.0.0 (190) software (IBM, Armonk, NY, USA).

Descriptive statistics of participant characteristics were computed. The responses to each of the 21 items measuring smoking cessation support practices were presented as stacked bar charts.

Ordinal logistic regression analysis was performed with advice to stop smoking as the dependent variable and age, sex (male vs. female), years of healthcare experience, department type (somatic vs. psychiatric), type of clinic (outpatient vs. inpatient vs. A&E/intensive care vs. other), and HCP type (nurse vs. physician vs. healthcare assistant vs. other) as independent variables. The model assumption of proportional odds was assessed by testing for parallel lines. P values < 0.05 were considered to be statistically significant.

## Results

In the period from June to September 2021, all clinical staff at Lillebaelt Hospital (3530 HCPs at somatic department and 468 HCPs at psychiatric department) received the survey link via their work email account, with two reminders sent to non-completers. A total of 1851 (response rate 46.3%) HCPs completed the survey, of whom 1645 (88.9%) responded that they had regular patient contact and were included in subsequent analyses. See Table 1 for an overview of participant characteristics.

### Non-responder characteristics

Of 3998 employees, 2147 (53.7%) did not respond to the survey after having received the initial survey link plus two reminders. Owing to anonymity in data collection, the specific characteristics of individual non-responders could not be obtained. However, the mean age of all employees at the included hospitals was 42.9 years and 86.5% were female. In terms of profession, 18.7% were physicians, 53.2% were nurses, 7.2% were healthcare assistants and 20.9% were of other professions.

**Table 1:** Participant characteristics

	Total (N = 1645)
Age, mean (SD), y	44.3 (11.8)
Sex, No. (%)	
Male	219 (13.3)
Female	1408 (85.6)
Healthcare experience, mean (SD), y	17.2 (12.0)
Department type, No. (%)	
Somatic	1522 (92.5)
Psychiatric	123 (7.5)
Type of clinic, No. (%)	
Outpatient clinic	534 (32.5)
Inpatient bed unit	589 (35.8)
A&E/intensive care	250 (15.2)
Other	221 (13.4)
HCP type	
Nurse	929 (56.5)
Physician	295 (17.9)
Healthcare assistant	139 (8.4)
Other*	222 (13.5)

*‘Other’ consists of physiotherapists, occupational therapists, psychologists, social workers, pedagogues, and students

A&E, Accident and Emergency; HCP, healthcare provider.

### Assessment of smoking

**Fig. 1:**
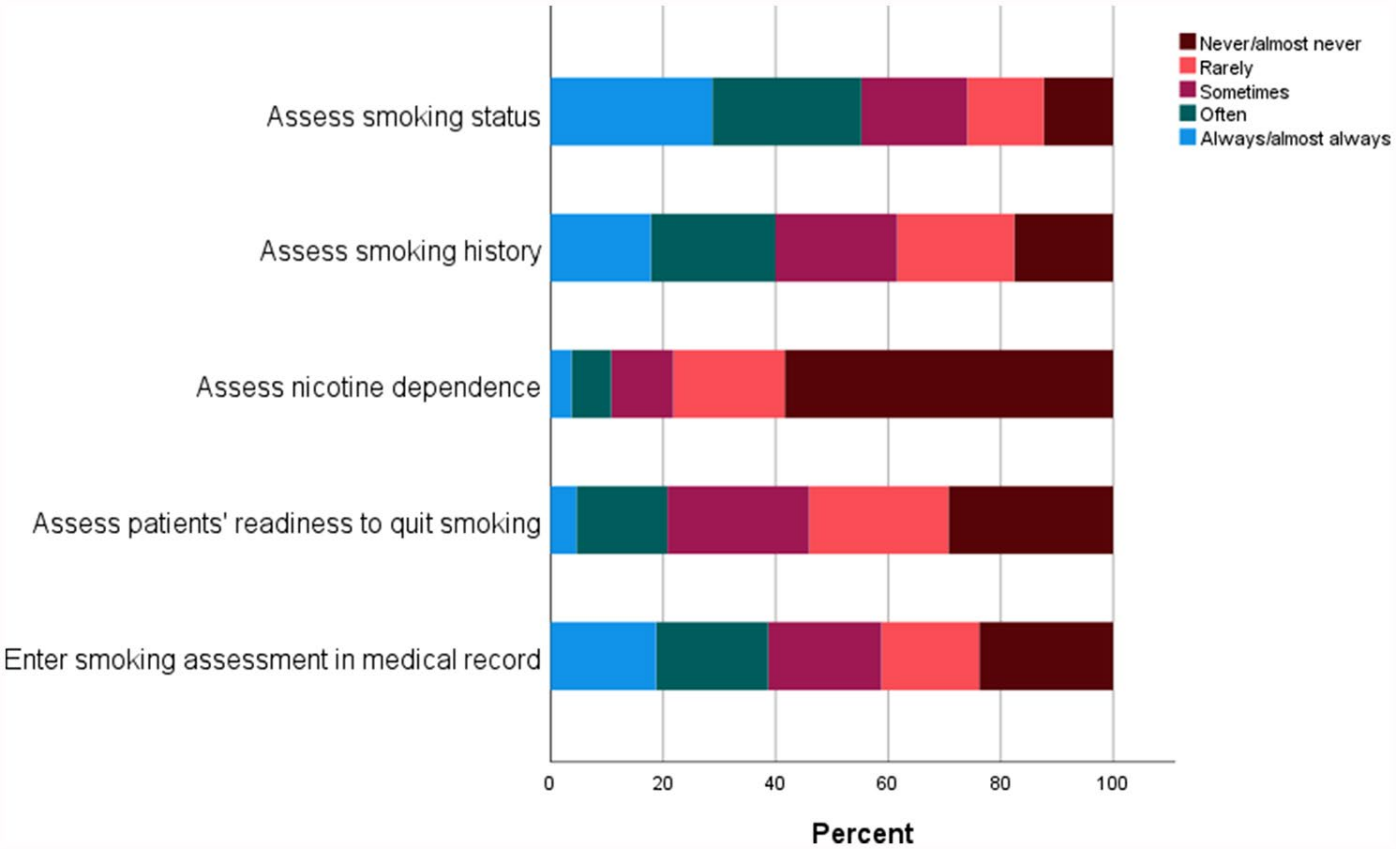
Healthcare providers’ responses to items related to the assessment of smoking

In total, 25.9% of HCPs reported that they never or rarely assess current smoking status, and 38.4% reported to never or rarely asses smoking history. Moreover, 78.2% reported that they never or rarely assess nicotine dependence, and 54.0% reported that they never or rarely assess patients’ readiness to quit smoking. With regard to documentation, 41.2% of HCPs reported that they never or rarely enter the results of the smoking assessment in patients’ medical files (see Fig. 1).

### Providing information and advice about smoking

**Fig. 2:**
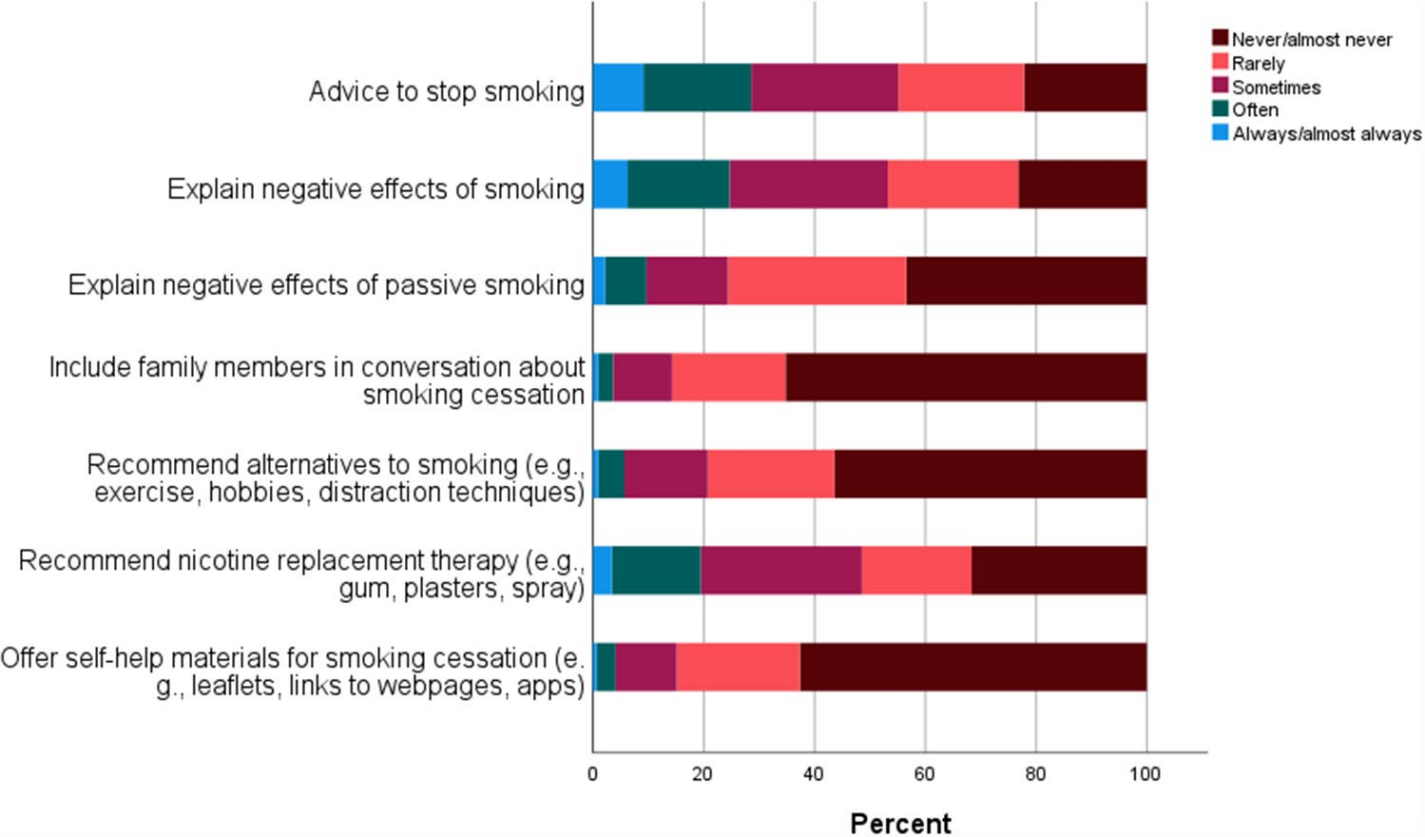
Healthcare providers’ responses to items related to information and advice about smoking

Figure 2 provides an overview of HCPs’ responses to items on information and advice about smoking. It was reported that 44.8% never or rarely advice smoking cessation, and 46.7% never or rarely explain negative effects of smoking. Furthermore, 75.6% of HCPs never or rarely explain the negative effects of passive smoking, and 85.7% never or rarely include family member in conversations about smoking cessation. Concerning advice on management strategies, 79.3% never or rarely recommend behavioural alternatives to smoking, and 51.4% never or rarely recommend nicotine replacement therapy. Lastly, 84.8% of HCPs reported that they never or rarely offer self-help materials for smoking cessation.

### Planning and referral to further support

**Fig. 3:**
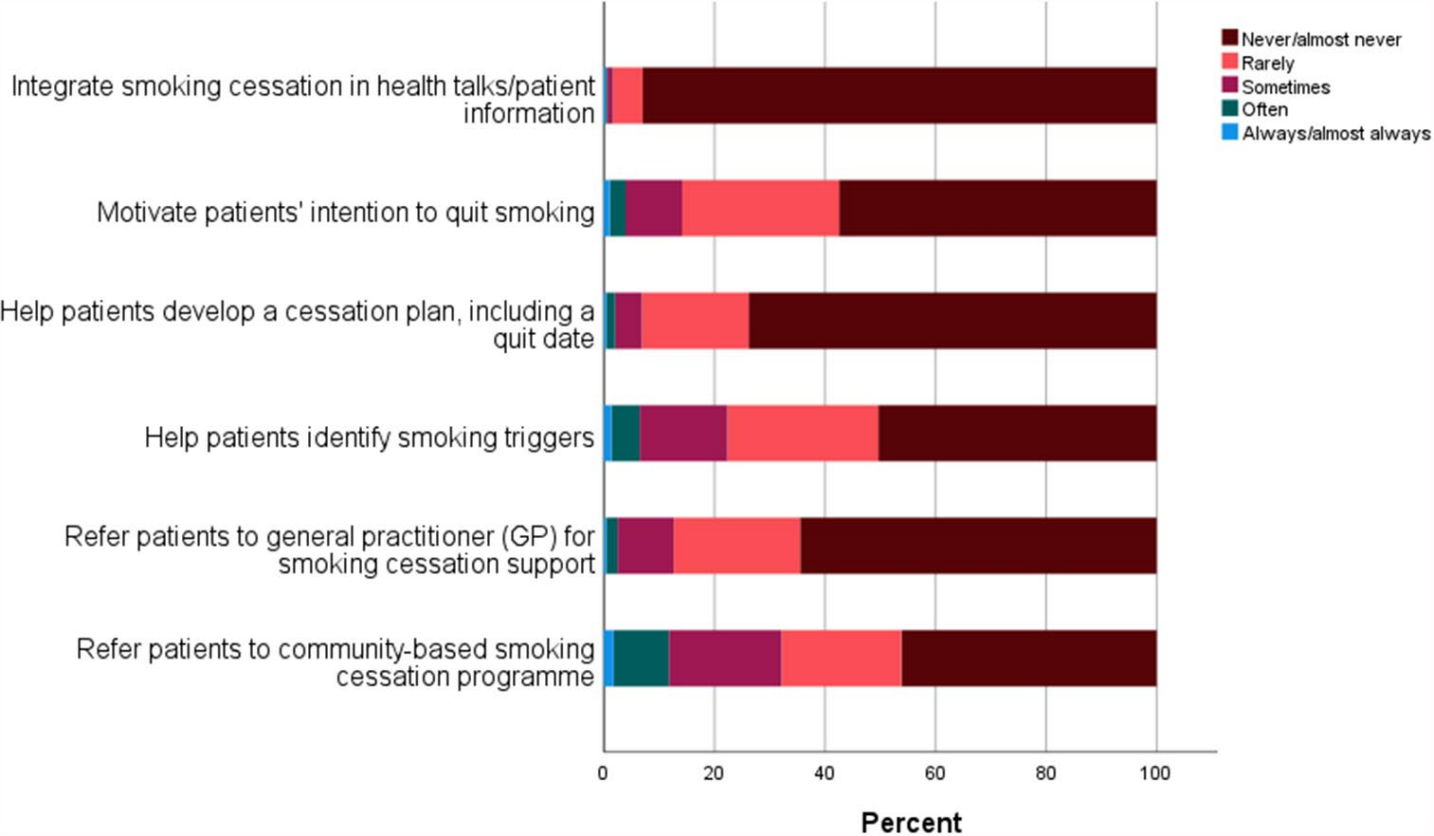
Healthcare providers’ responses to items related to planning and referral to further support for smoking cessation

An overview of HCPs’ responses to items on planning and initiation of smoking cessation and referral to further smoking cessation support can be found in Fig. 3. It was reported that 98.4% of HCPs never or rarely integrate smoking cessation support in hospital-based patient information meetings or health talks. Furthermore, 85.5% reported that they never or rarely seek to stimulate patients’ motivation to stop smoking, and the majority reported that they never or rarely help patients develop a cessation plan (93.1%) or identify triggers for smoking (77.6%). Concerning referral to non-hospital-based smoking cessation support, 87.4% of HCPs reported that they never or rarely refer patients to their GP, and 67.9% never or rarely refer patients to community-based smoking cessation programmes.

### Follow-up on smoking cessation

**Fig. 4:**
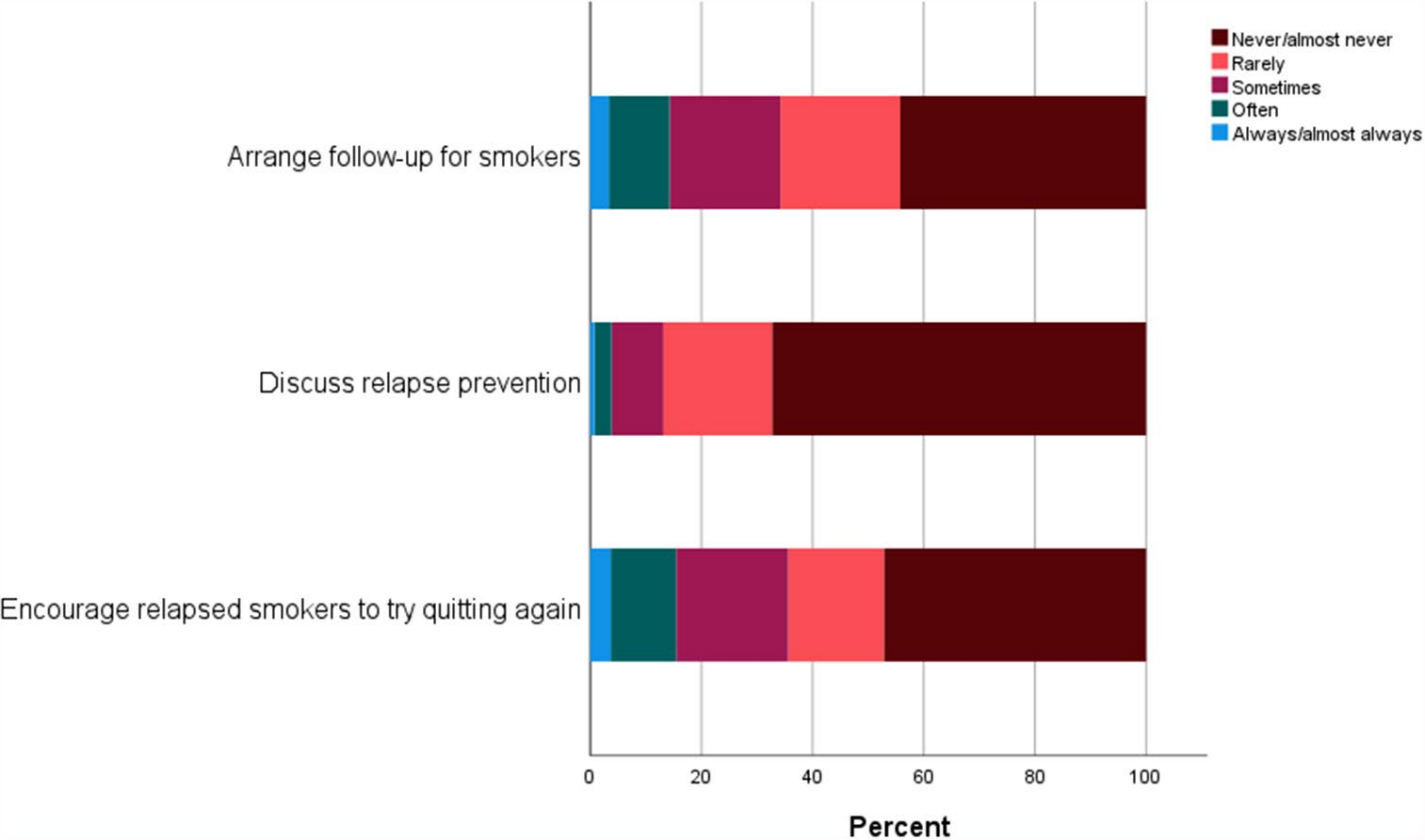
Healthcare providers’ responses to items related to follow-up on smoking cessation

Figure 4 gives an overview of HCPs’ responses to items on follow-up. Sixty-six per cent reported that they never or rarely arrange follow-up for smokers, and 86.8% reported that they never or rarely discuss relapse prevention with previous smokers. Furthermore, 64.4% reported that they never or rarely encourage relapsed smokers to try quitting again.

### Predictors of HCPs giving advice on smoking cessation

The results of the ordinal regression analyses can be found in Table 2. The model met the assumption of proportional odds (p = 0.063). HCPs in inpatient departments were 31% less likely to give advice on smoking cessation than HCPs in outpatient departments (odds ratio [OR] 0.69, 95% confidence interval [CI] 0.55–0.88), and HCPs in A&E were 55% less likely than HCPs in outpatient departments (OR 0.45, 95% CI 0.34– 0.61). HCPs in other clinic types were 81% less likely to give advice on smoking cessation than HCPs in outpatient departments (OR 0.19, 95% CI 0.14–0.26). Furthermore, physicians were more than three times as likely to advise on smoking cessation than nurses (OR 3.55, 95% CI 2.67–4.71). No statistically significant effects of age, sex, years of experience or department type (psychiatric vs. somatic) were found.

**Table 2:** Predictors of giving advice on smoking cessation among hospital-based healthcare providers

Predictor	OR (95% CI)	*p*
Age	0.999 (0.981–1.016)	0.871
Sex		
Female	-	
Male	1.215 (0.853–1.731)	0.281
Healthcare experience	1.010 (0.992–1.027)	0.271
Department type		
Psychiatric	–	–
Somatic	1.215 (0.853–1.731)	0.281
Type of clinic		
Outpatient clinic	–	–
Inpatient bed unit	0.694 (0.54–0.878)	**0.002**
A&E/intensive care	0.454 (0.339–0.608)	**< 0.001**
Other	0.189 (0.138–0.260)	**0.000**
HCP type		
Nurse	–	–
Physician	3.545 (2.669–4.708)	**0.000**
Healthcare assistant	0.820 (0.575–1.170)	0.273
Other	0.771 (0.578–1.029)	0.077

*p* values in bold are statistically significant at the 0.05 level.

A&E, Accident & Emergency unit; HCP, healthcare provider.

## Discussion

We carried out a survey of smoking cessation support practices among 1645 HCPs, comprising a representative sample of employees from a hospital, including outpatient and inpatient settings. Generally, the results indicated that the existing practices for supporting smoking cessation in the hospital are limited, underlining the gap between the strong existing evidence for smoking cessation support and the actual implementation of such interventions in practice [28].

### Assessment of smoking

The results of this study showed that more than 25% of included HCPs never or rarely ask patients about their smoking status. Moreover, smoking history, nicotine addiction and motivation to quit smoking were only infrequently assessed by HCPs in the present study, despite being important information to include in a subsequent referral to a smoking cessation programme, with the purpose of determining a specific quit date [29] or planning types and delivery of nicotine substitution [30]. With regard to documentation, more than 40% of the HCPs in the present study reported that they never or rarely enter the results of the smoking assessment in patients’ medical files. This finding could be explained by the fact that one patient often has contact with more than one HCP during a hospital visit, and only one HCP is required to perform smoking assessment per visit. But if none of the dedicated HCPs performs a smoking assessment and enters the information in the patient record, it leaves little information for other HCPs in the hospital system to act on, which is problematic for the team approach to the problem [31]. Moreover, it prevents the hospital management from monitoring and following up on initiatives to increase smoking cessation support.

The limited smoking assessment and documentation is problematic, as it contradicts the existing clinical guidelines [32]. For example, for any procedure that involves anaesthesia, smoking increases the risk of surgical complications [33]. Specifically in lung cancer, smoking increases the risk of postoperative morbidity and mortality [34], and smoking history is therefore an important element in the preoperative risk assessment. In the psychiatric setting, standard assessment of smoking habits is also relevant (e.g. changes in tobacco use may interact with certain antipsychotic drugs) [35].

The results of the present study showed that almost half of participating HCPs never or rarely advise on smoking cessation, or explain the negative effects of smoking. In terms of specific approaches to information provision, the majority reported that they never or rarely use the supportive interventions of including family members; recommending behavioural alternatives to smoking; recommending nicotine replacement therapy; or self-help materials. The low levels of smoking cessation support in the hospital are worrying because hospital-based HCPs are often trusted and seen as important authorities when it comes to making health-related decisions. For example, a recent national survey on smoking habits in Denmark showed that thoughts about health was the most common factor (69%) contributing to a quit attempt for respondents who tried to stop smoking within the last year [36]. Ten per cent reported that encouragement from HCPs was a contributing factor to their most recent attempt [36]. As thoughts about health and communication with HCPs are inherent in hospital visits, the hospital setting is an optimal place to provide information on the negative health consequences of smoking and provide advice to stop. This resonates with the findings of a recent study suggesting that direct advice on smoking cessation from HCPs helped patients move from the contemplation and preparation stages to the action stage in their smoking cessation process [12]. The tendency for nicotine substitution therapy to show a limited effect when provided without counselling from a HCP is another important argument for delivering smoking cessation support during the ‘window of opportunity’, when patients are already in contact with the hospital [37].

When exploring possible predictors of providing advice to stop smoking, the results of the present study showed that HCPs working in inpatient units (including A&E and intensive care units) were less likely to advise smoking cessation than HCPs working in outpatient units. There could be many explanations for these findings, including organizational structures such as consultation time, number of consultations and different HCPs delivering the consultations, combined with the fact that many hospitals face challenges of increased healthcare demands and limited personnel. However, the limited practice of smoking cessation support found in the present study could also be influenced by the psychological processes of individual employees (e.g. knowledge and beliefs about smoking, and self-efficacy), as well as social and cultural norms within the organization [38]. A recently published study based on focus groups with hospital-based HCPs revealed that time constraints and competing priorities in clinical settings act as barriers to providing smoking cessation support [39]. HCPs have expressed a need for further training in communication skills and an update on best practice in smoking cessation support [39].

The results of the present study showed that physicians were more likely than nurses to advise on smoking cessation. This result may be an expression of a classic labour division between physicians and nurses, where the physician gives brief advice to stop smoking, which can done relatively quickly and easy, while the nurses, who act as care managers, engage in conversations with patients about motivation and psychosocial barriers to stopping smoking, which require more time and training [40]. While differences across HCP types in giving advice to stop smoking may be partially explained by labour division in specific hospital departments in the present study, it should be highlighted that smoking cessation support is a shared professional responsibility, and the likelihood of sustained smoking abstinence increases with repeated advice from multiple HCPs across clinical contexts [19–21]. It is possible that the HCP types in the present study are too general, and there may be differences within each HCP category (e.g. between nurses in respiratory vs. paediatric settings) [41], which are not reflected in the present results. Future studies with more detailed information on specific care units are needed to shed light on potential differences. In the present study, differences in department type (psychiatric vs. somatic), age, sex, and HCPs’ years of experience did not affect the provision of advice to stop smoking.

The majority of the HCPs in the present study reported that they never or rarely refer patients to either community- or GP-based smoking cessation support, despite local guidelines and e-learning programmes instructing them to do so [42, 43]. At the same time, the majority of HCPs in the present study reported that they never or rarely initiate any planning activities themselves, for example in the form of integrating smoking cessation support in patient information materials; motivating patients to stop smoking; helping patients develop a cessation plan; or helping patients to identify smoking triggers. These findings mirror the general tendency of an increasing number of smokers in European countries quitting without the help of HCPs, while the use of supportive interventions such as pharmacotherapy and smoking cessation services declined in the period from 2012 to 2017 [22].

The provision of smoking cessation support has previously been presented in a pyramid model with different levels of care, ranging from a general level, where all smokers are influenced by public health campaigns and policies, to the most specialized level, where individuals with high levels of addiction and several failed quit attempts receive pharmacological treatment and cognitive behavioural therapy in specialized smoking cessation clinics [13]. At the intermediate levels in the pyramid, HCPs refer individuals to more specialised levels of smoking cessation interventions. Hence, HCPs in hospitals play an important role as gatekeepers for the referral of patients to more specialized smoking cessation support.

In a recent study from the Netherlands [40], factors related to HCPs (limited) referral behaviour may be understood based on the COM-B model, in which behaviour (B) is generated by the components of capability (C), opportunity (O) and motivation (M) [44]. Their results indicated that the ability to refer patients to smoking cessation support was challenged by a lack of knowledge about specific referral options and procedures; that HCPs regarded their consultation as an opportunity for initiating smoking cessation, but, at the same time, left patients with the responsibility to take the next step; and that HCPs’ motivation to refer patients to counselling outside their own practice was low because the quality and trustworthiness of the receiving clinic was unknown.

The results of the present study showed that the majority of included HCPs reported that they never or rarely arrange follow-up, discuss relapse prevention or encourage relapsed smokers to try quitting again. Historically, smoking cessation has been considered an *event* that could lead to either success (no smoking) or failure (continued smoking). However, during the last 50 years, alongside general developments in behaviour change theories, smoking cessation has come to be seen as a *process* that includes a smaller or larger number of quit attempts, as described in the transtheoretical model of behaviour change [45]. From this perspective, the relapse and maintenance stages in the smoking cessation process include experiences (e.g. recognizing smoking triggers and seeking help in spite of feelings of guilt) that are just as important leaning points for prolonged smoking abstinence as experiences in the planning and action phases (e.g. setting a quit date and managing the urge to smoke). Smoking should be considered a chronic condition with a life-long risk of relapse, and should therefore be considered a relevant aspect of all healthcare visits [32].

The results of the present study highlight the need to improve hospital-based practice of smoking cessation support in the future. A recent review of implementation studies indicated that staff training is the predominant approach to achieving this [46]. In the present study, nurses are less likely to advise smoking cessation than physicians, although advice could be delivered as an integral part of other care tasks that are usually delivered by nurses (e.g. general patient education, psychosocial support, wound care and medicine dispensing). Training HCPs in how to provide smoking cessation support as part of routine care could be an important element in optimizing hospital-based smoking cessation support. Implementation approaches should never rely on a single component, and planning, resourcing and investing in a multi-strategic approach is essential to drive successful implementation [46]. Furthermore, smoking cessation usually proceeds over a longer period of time than the hospital course, and successful smoking cessation support in the hospital setting should therefore not be determined by the number of patients who stop smoking during their hospital visit or stay. Instead, the primary implementation outcome could be patients’ motivation to quit; their perceived self-efficacy; or the number of patients that are referred to specialized smoking cessation programmes, while quit rates could be applied as a secondary, longer-term implementation outcome.

This study assessed the implementation outcome of *adoption* (i.e. whether staff take action to support smoking cessation) [46]. Assessment of other outcomes, such as cost, acceptability and appropriateness, would have provided a more detailed look into the implementation of smoking cessation support in the clinic but would require other data sources than self-reported survey data alone.

The present study is among the first to explore practices of smoking cessation among HCPs in one hospital. It was based on a large, representative sample of employees, and was performed independently and anonymously, increasing the reliability and reducing bias in the results.

Meanwhile, a number of limitations should be noted. Firstly, data were collected from the provider perspective and were solely based on self-report, compromising the external validity of the findings. Future studies should use triangulation approaches in the data collection by obtaining results on the patient level, the provider level and the organizational level. Secondly, the response rate was 46.3%, and, due to anonymity of data collection, the individual characteristics of non-responders could not be obtained. Nonetheless, the characteristics of the survey completers were generally comparable with the general characteristics of employees at the hospital. Thirdly, smoking infrastructure (e.g. community- or hospital-based placement of specialized smoking cessation programmes) is different across countries, and it may therefore not be possible to generalize the results of the present study to hospital settings in other countries.

## Conclusion

The results of our large-scale survey of HCPs in one hospital show that smoking cessation support is limited in the areas of smoking assessment; providing information and advice; planning and referral to further support; and follow-up on smoking cessation attempts. HCPs working in inpatient units were less likely to advise smoking cessation than those working in outpatient clinics, and physicians were more likely to advise smoking cessation than nurses. The results of the present study underline the gap between the strong existing evidence for smoking cessation support and the implementation of such interventions in practice. There is a need for future studies to evaluate the cost-effectiveness, acceptability and appropriateness of implementation strategies to optimize smoking cessation support in the hospital setting. Collecting data at the patient, provider and organization levels will increase external validity in future studies.

## Electronic supplementary material

Below is the link to the electronic supplementary material.

Supplementary Material 1: Checklist for Reporting of Survey Studies (CROSS)

Supplementary Material 2: Survey items

## Data Availability

The datasets used and/or analysed during the current study are available from the corresponding author on reasonable request.

## Abbreviations

A&E: Accident and Emergency
GP: General practitioner
HCP: Health care provider
OR: Odds ratio
SD: Standard deviation
UK: United Kingdom
