# Supplementary material for: A survey exploring the practices of smoking cessation support among hospital-based healthcare providers
Source: BMC Health Serv Res. 2023 Jun 16;23:645. doi: 10.1186/s12913-023-09657-4 (PMC10276475; doi:10.1186/s12913-023-09657-4)
Supplement: Supplementary file 2 — Supplementary Material 2: Survey items [file 12913_2023_9657_MOESM2_ESM.docx]

| **Question** | **Options** | **Comments** |
| --- | --- | --- |
| ***Introduction*** | | |
| Dear participant  In this survey, we want to investigate to what extent and in which way patients are currently being informed about smoking cessation and how they are supported at the department where you are employed.  We will ask you to answer a series of questions regarding your personal experiences and practices in relation to providing information and/or providing support for smoking cessation for  patients at the department.  **The survey consists of 44 questions and takes approx. 10-15 minutes to complete**. **Your answer will be used for research purposes as well as to identify factors of relevance for smoking cessation support in clinical practice. Your individual responses will *not* be shared with manager, colleagues, patients or others outside the research group.**  Many thanks for your participation.  Sincerely,  the research group at the Department of Medicine at Lillebaelt Hospital, Vejle and the Department of Psychiatry, Vejle |  |  |
| ***Socio-demographic and work-related variables*** | | |
| Does your position involve patient contact in the form of treatment and/or care? | Yes  No |  |
| How old are you? (please write your age in the field below) | Number field |  |
| Which sex are you? | Female  Male |  |
| Which department do you work in? | Departments listed (select one) |  |
| What type of clinic do you mainly work in? | Outpatient clinic  Inpatient bed unit  Accident/Emergency (A&E)  Intensive are  Other |  |
| For how long have you been employed at your current workplace?  (Indicate number of years. If you have been employed for less than one year, please enter 0 in the answer field) | Number field | Not included in the present study |
| What do you work as at your current workplace? | Nurse  Physician (incl specialists)  Healthcare assistant  Social worker  Physiotherapist  Occupational therapist  Psychologist  Pedagogue  Student  Other |  |
| How many years of experience have you accumulated within the health field? (Indicate number of years. If you have less than one year of experience, please write 0 in the answer field) | Number field |  |
| ***Practice of smoking cessation support*** | | |
| How often do you assess patients' current smoking status? | Always/almost always  Often  Sometimes  Rarely  Never/almost never | Assessment of smoking (≈’Ask’ in Chan et al.*) |
| How often do you assess patients' smoking history? (e.g. how many years they have smoked; how many cigarettes they have smoked daily; previously smoke free periods) | Always/almost always  Often  Sometimes  Rarely  Never/almost never | Assessment of smoking (≈’Ask’ in Chan et al.*) |
| How often do you assess patients’ nicotine addiction? (e.g. via Fagerström's test or detection of withdrawal symptoms) | Always/almost always  Often  Sometimes  Rarely  Never/almost never | Assessment of smoking (≈’Ask’ in Chan et al.*) |
| How often do you enter the results of smoking assessment in patients' medical record? | Always/almost always  Often  Sometimes  Rarely  Never/almost never | Assessment of smoking (≈’Ask’ in Chan et al.*) |
| How often do you talk to patients who smoke about the negative effects of smoking? | Always/almost always  Often  Sometimes  Rarely  Never/almost never | Providing information and advice (≈’Advice’ in Chan et al.*) |
| How often do you talk to patients and/or their relatives about the negative effects of passive smoking? | Always/almost always  Often  Sometimes  Rarely  Never/almost never | Providing information and advice (≈’Advice’ in Chan et al.*) |
| How often do you advise patients who smoke to stop smoking? | Always/almost always  Often  Sometimes  Rarely  Never/almost never | Providing information and advice (≈’Advice’ in Chan et al.*) |
| How often do you have motivational conversations about smoking cessation with patients who smoke? | Always/almost always  Often  Sometimes  Rarely  Never/almost never | Planning and referral (≈’Advice’ in Chan et al.*) |
| How often do you help patients who smoke to identify the factors that play a role in maintaining their smoking (smoking triggers)? (e.g. smoking in order to  calm stress/nervousness, cozy smoking with friends etc.) | Always/almost always  Often  Sometimes  Rarely  Never/almost never | Planning and referral  (≈’Assess’ in Chan et al.*) |
| How often do you examine patients' readiness to quit smoking? | Always/almost always  Often  Sometimes  Rarely  Never/almost never | Assessment of smoking  (≈’Assess’ in Chan et al.*) |
| How often do you help patients who smoke to develop a smoking cessation plan, including a quit date? | Always/almost always  Often  Sometimes  Rarely  Never/almost never | Planning and referral  (≈’Assist’ in Chan et al.*) |
| How often do you refer patients who smoke to a community-based smoking cessation program? | Always/almost always  Often  Sometimes  Rarely  Never/almost never | Planning and referral  (≈’Assist’ in Chan et al.*) |
| How often do you refer patients who smoke to their general practitioner (GP) for smoking cessation support? | Always/almost always  Often  Sometimes  Rarely  Never/almost never | Planning and referral  (≈’Assist’ in Chan et al.*) |
| How often do you offer relevant self-help material to patients who smoke? (e.g. in the form of pamphlets, links to internet sites/ telephone counselling / app etc.) | Always/almost always  Often  Sometimes  Rarely  Never/almost never | Providing information and advice  (≈’Assist’ in Chan et al.*) |
| How often do you recommend alternatives to smoking for patients who smoke? (e.g. physical activity, hobbies, breathing exercises, distraction techniques etc.) | Always/almost always  Often  Sometimes  Rarely  Never/almost never | Providing information and advice  (≈’Assist’ in Chan et al.*) |
| How often do you recommend nicotine replacement therapy for patients who smoke? (e.g. nicotine patch/chewing gum/spray etc.) | Always/almost always  Often  Sometimes  Rarely  Never/almost never | Providing information and advice  (≈’Assist’ in Chan et al.*) |
| How often do you include family members in coversations about smoking cessation (with the patient's consent)? | Always/almost always  Often  Sometimes  Rarely  Never/almost never | Providing information and advice  (≈’Assist’ in Chan et al.*) |
| How often do you integrate smoking cessation in health takes/patient information? | Always/almost always  Often  Sometimes  Rarely  Never/almost never | Planning and referral  (≈’Assist’ in Chan et al.*) |
| How often do you arrange follow up on patients' attempts to quit smoking? | Always/almost always  Often  Sometimes  Rarely  Never/almost never | Follow-up  (≈’Arrange’ in Chan et al.*) |
| How often do you discuss relapse prevention with patients who attempts to quit smoking? | Always/almost always  Often  Sometimes  Rarely  Never/almost never | Follow-up (≈’Arrange’ in Chan et al.*) |
| How often do you support/encourage patients who have relapsed to smoking to quit again? | Always/almost always  Often  Sometimes  Rarely  Never/almost never | Follow-up (≈’Arrange’ in Chan et al.*) |
| ***Skills, ressources and motivation*** | | |
| To what extent do you have the necessary skills to deliver smoking cessation support? | 0: Not at all  1  2  3  4  5  6  7  8  9  10: To a great extent | Not included in the present study |
| To what extent do you have the necessary resources at your workplace  (e.g. time, premises , etc. ) to carry out smoking cessation advice, which is available  beyond the VBA method? | 0: Not at all  1  2  3  4  5  6  7  8  9  10: To a great extent | Not included in the present study |
| To what extent are you motivated to undertake smoking cessation counseling,  which is beyond the VBA method? | 0: Not at all  1  2  3  4  5  6  7  8  9  10: To a great extent | Not included in the present study |
| Have you received any training in smoking cessation counseling? | Yes  No | Not included in the present study |
| If you have received education, what kind of education  have you received it? (you are welcome to tick more than one, if relevant) | Qualified smoking cessation counselor via the Danish Cancer Society (3-day course)  Qualified smoking cessation counselor via a provider other than the Danish Cancer Society (course on more than 1 day)  Received shorter training, e.g. at work or elsewhere (max. 1 full day)  Acquired knowledge myself (via internet/ pamphlets/ books)  Other | Not included in the present study |
| Do you experience a need for (further) training in  smoking cessation advice? | Yes  No | Not included in the present study |
| ***Barriers***** | | |
| Tick what is preventing you from supporting  patients' smoking cessation. (You are welcome to tick more than one)  Lack of knowledge on how to support smoking cessation  Lack of skills  Lack of time  Lack of support (e.g. from colleagues/hospital admin)  Lack of resources (e.g. rooms, information materials)  Lack of backing from management  Heavy or overwhelming workload  Lack of patient motivation (e.g. does not want to stop)  Lack of confidence in my own abilities  Personal discomfort  Unwilling to upset the patient  Unwilling to risk damage to patient alliance  Smoking cessation is not a priority  Lack of own motivation  Sceptical of the effectiveness of smoking cessation support  Lack of incentive (e.g. smoking cessation advice is not recognised/rewarded at my workplace)  Negative past experiences with smoking cessation support  Social/peer pressure  Smoking viewed as a coping mechanism for patients  Lack of knowledge about how to refer patients to community-based smoking cessation program  Lack of knowledge about contents and quality of community-based smoking cessation program that patients are referred to  I experience NO barriers | Response categories reported in the left coloumn | Not included in the present study |
| What other barriers do you experience? (Please describe below) | Free-text field | Not included in the present study |
| ***Additional comments*** | | |
| Please write below if you have any comments on the survey and/or want to elaborate on some of your answers? | Free-text field | Not included in the present study |
| **Thank you very much for your participation!**  If you have questions or comments about the survey and are employed by psychiatry, you are very welcome to contact the project coordinators. |  |  |

*Based on specific Five A-questions (Ask, Advise, Assess, Assist, Arrange) developed by Chan SSC, Sarna L, Wong DCN, Lam TH. Nurses’ Tobacco-Related Knowledge, Attitudes, and Practice in Four Major Cities in China. J Nurs Scholarsh 2007;39(1):46–53.

**The response categories are based on the clinican-reported barriers in the systematic review by Sharpe T, Alsahlanee A, Ward KD, Doyle F. Systematic Review of Clinician-Reported Barriers to Provision of Smoking Cessation Interventions in Hospital Inpatient Settings. J Smok Cessat 2018;13(4):233–243.
